# The Effect of Household Food Processing on Pesticide Residues in Oranges (*Citrus sinensis*)

**DOI:** 10.3390/foods11233918

**Published:** 2022-12-05

**Authors:** Perihan Yolci Omeroglu, Busra Acoglu Celik, Elif Koc Alibasoglu

**Affiliations:** 1 Department of Food Engineering, Faculty of Agriculture, Gorukle Campus, Bursa Uludag University, Bursa 16059, Turkey; 2 Science and Technology Application and Research Center (BITUAM), Gorukle Campus, Bursa Uludag University, Bursa 16059, Turkey

**Keywords:** pesticide residues, orange, household food processing, processing factor

## Abstract

In this study, the effect of various household food-processing methods (washing, peeling, processing into jam and fruit juice, freezing, storage) on pesticide residues (abamectin, buprofezin, ethoxazole, imazalil, and thiophanate-methyl) in oranges was investigated. Residue analyses were performed by quick-easy-cheap-efficient-rugged-safe (QuEChERS) extraction and liquid chromatography coupled with triple quadrupole mass spectrometry (LC-MS/MS) analysis. The limit of quantification of the method for each pesticide was 10 µg/kg. Physicochemical properties of the pesticides and the type of the food process had a considerable effect on the fate of pesticide residue. Pesticide residues were mostly dispersed on orange peels and washing with tap water decreased the residue levels by 26–84%. The amount of residue in oranges was reduced by 63–100% during fruit juice processing, while residues were removed by 90–100% after jam processing. Pesticides with a high octanol–water coefficient were absorbed by the wax of the orange peel, therefore they remained on the peel and could not easily be removed by washing. Moreover, pesticides with lower water solubility did not diffuse easily through the fruit juices from the pulp section of the fruit. The processing factor was greater than 1 for the separation of the orange peel and less than 1 for the washing step and jam and fruit juice productions.

## 1. Introduction

Orange (*Citrus sinensis*) is one of the most popular and most-grown citrus families in the world. According to the Turkish Mediterranean Exporters’ Association, the citrus group took first place in Turkish total fruit and vegetable exports between 2017 and 2018 (405.2 million tons) [1]. Valencia is the most important orange variety grown in Turkey, and its farming has been increasing rapidly in recent years. Its most important feature is that it can be grown and harvested up to the late months of the spring. The peel of the fruit is slightly rough and moderately thick and the inner skin is thick and contains few seeds. Orange is a good source of vitamins, flavonoids, terpenes, potassium, and calcium, and fulfills most of the daily need for vitamin C for consumers. Orange can be consumed fresh or processed domestically or industrially into jam, marmalade, or fruit juice, in addition to its frozen and dried forms [2]. Orange peel is used as a flavoring agent in the pastry sector and essential oils extracted from the peels are used in the cosmetics industry.

Since citrus fruits are cultivated in warm and subtropical climates where unwanted pests and wild herbs are common, it is normal agricultural practice to use pesticides to increase the yield of the crop [3]. However, excessive and unprescribed spraying and early harvesting can leave residues in the product, which may adversely affect the safety of the food consumed. Pesticide residue levels in foods must be safe for consumers, so national and international authorities have set maximum residue limits (MRL) for fresh fruits and vegetables, animal products, and feeds [4] (EC 2005). Processing factor (*P*_F_) is defined as the ratio of pesticide residue level in processed food to the pesticide residue level in the raw agricultural commodity (RAC). Processing factors are taken into account when verifying the compliance of residues in processed products with the MRL of agricultural commodities and to refine dietary exposure intake with respect to residues in processed products [5,6,7]. The studies reported in the literature revealed that pesticide residues in RACs can be decreased or increased as a result of the processes applied during the preparation of the products for consumption both at industrial and household-scale productions, including washing, peeling, freezing, heating, and drying [8,9,10,11].

Although oranges and their products are widely consumed throughout the world, the fate of the pesticide residues during the processing of oranges has been revealed in a limited number of studies in the literature [2,3,12,13,14]. The panel of experts on Pesticide Residues in Food and the Environment and the WHO Core Assessment Group on Pesticide Residues reported processing factors for different type of pesticides in food commodities. The reports included processing factors of pasteurized or canned orange juice, canned orange marmalade, dried orange peel oil, dried orange pulp, and chopped fresh orange peel, for different type of pesticides including buprofezin, dimethoate fenpyroximate, fluensulfone, imizalil metalxyl, mandipropamid, omethate, and pydiflumetofen [14]. Since processing effects depend on the physicochemical properties of the pesticide in addition to the type of the process and the matrix, processing factors should be individually determined for each pesticide, process, and matrix combination [5,15]. Therefore, there is still a need for further studies on the other common pesticides and household processing of oranges. In the light of the needs in the literature, we aimed to study the effects of various household food processing techniques (washing with tap water, peeling, pulping, processing to jam or fruit juice, storage of frozen grated peels for a certain period of time) on the initial concentration of abamectin, buprofezin, etoxazole, imazalil, and thiophanate-methyl residues in oranges. The pesticides were selected among the authorized commercial pesticides commonly used for local farming applications of citrus in Turkey.

## 2. Materials and Methods

### 2.1. Sampling

The oranges used in the study were purchased from the local market in Bursa in 2018, it was indicated that oranges were cultivated from Antalya region in Turkey in the same year [2].

Prior to analysis, samples were kept in the cold storage room at 5–7 °C and 90–95% relative humidity. Before pesticide treatment at laboratory conditions, three laboratory samples (C) were allocated as blank samples. It was found that control samples contained only one pesticide (imazalil) at 0.01 mg/kg level [2].

The moisture level of the control samples “C” was measured as 94.05% (MA150 Sartorius, Göttingen, Germany). The weights of each orange unit in laboratory sample “C” varied between 223 g and 246 g. Therefore, orange samples were defined as medium-sized products according to legal regulations. In this manner, in total 30–35 kg of orange samples were used, to make approximately 1 kg laboratory samples (at least 10 units for raw orange samples) depending on the type of the process [16].

### 2.2. Pesticide Treatment

The orange samples used in the scope of this study were immersed in to the solutions of the commercial formulations at the laboratory [17,18,19]. Therefore, a homogeneous distribution was provided between and within each unit of laboratory samples and a detectable amount of residue was obtained [2].

**Figure 1:**
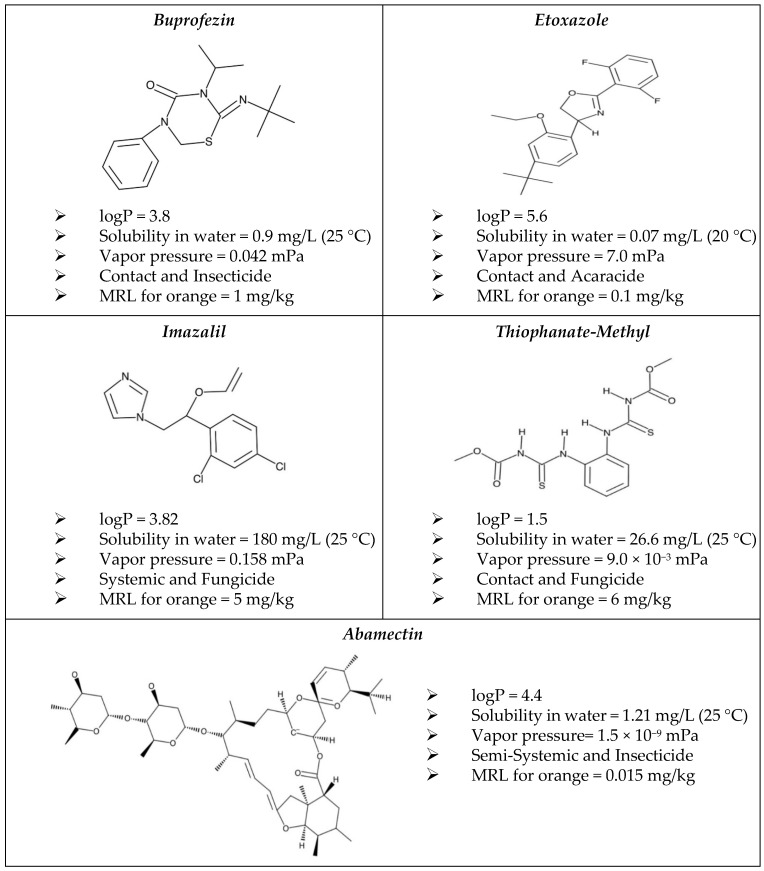
Physicochemical structure and mode of action for the active ingredient (pesticide). MRL represents maximum residue level (EC 2005).

Commercial formulations of abamectin, buprofezin, etoxazole, imazalil, and thiophanate-methyl were purchased from a local seller. The physicochemical properties of the active ingredients were provided in Figure 1. The commercial formulations of the pesticides were Asmition (18 g/L, emulsified concentrate), Korfezin (400 g/L, suspension concentrate), Sorides (500 g/L, emulsified concentrate), Emtop (60%, wetted powder), and Novamite (110 g/L, suspension concentrate), respectively. Their recommended dose for 100 mL during good agricultural practices were 25 mL, 35–65 mL, 25 mL, 400 mL, and 60 g, respectively. To obtain pesticide residue levels in the raw agricultural commodity (RAC) higher than the limit of quantification of the method, it is permissible to apply more formulations than the recommended dose [15]. Therefore, approximately one to four times the recommended dose of the formulations (indicated on the prospectus) were taken and diluted in 10 L of water in a plastic containers (15–20 L) providing a homogenous distribution of the mixture. To treat all orange samples at the same time for 30 min, a proper number of the plastic containers with 10 L of mixture solutions were prepared. Subsequently, all samples were sun dried for 3–4 h. Three laboratory samples were allocated from the dried samples as “RAC” samples. From the remaining samples, three laboratory samples were separated for each of the processes as explained below. Dried samples were stored at +4 °C for 1 day until further analysis [2].

### 2.3. Household Processing

#### 2.3.1. Washing (W)

Washing was the initial step for all household processing applied in the scope of this study. Orange samples were rubbed by hand under flowing tap water at 10 °C for 1–2 min.

#### 2.3.2. Peeling: Separation of Peel (P) and Pulp (PU)

The peel and white flesh of the orange samples were separated from the fruit pulp with a knife. Peel/fruit weight ratios (%) ranged between 29% and 35%. The moisture content of the samples prior to peeling and the peel were 5.33 g H_2_O/g sample in dry base and 15.81 g H_2_O/ sample in dry base, respectively.

#### 2.3.3. Frozen Grated Peels (GP)

The peel separated from the fruit were grated and stored at −20 °C for three months. In order to investigate the effect of storage period on the fate of the residue, the amount of residue in the frozen grated peels were determined monthly.

#### 2.3.4. Fruit Juice (FJ)

Orange samples were divided into two sections to make fresh fruit juice by a homebased processer. The mean pH value of the juices was determined as 2.82 ± 0.05 (Mettler Toledo Seven compact pH/Ion pH meter, Colombus, OH, USA).

#### 2.3.5. Homemade Jam (HJ)

The jam production was based on a local recipe. The peels of the orange samples were gently grated and removed. In order to remove the bitterness of the outer thin layer on the fruit pulp, the pulps were boiled in water 3 times for 15 min. For each time, boiling water was replaced with fresh. Then, the fruits were sliced into 2-cm-thick cubes with a knife. Sugar syrup was prepared by dissolving sugar in water at 1:1 concentration (*w*/*w*) followed by boiling for 30 min. Orange juice (1 mL) was added to the syrup at the end of the boiling step. The fruits were added in to the syrup and cooked at 95 °C for 30 min until the jam reached a proper consistency. The hot jam was put into the glass jars. Subsequently, the jars were turned upside down for cap sterilizations and kept in this form until they were cool. pH of the jam was measured as 3.45 ± 0.06 and water-soluble dry matter (Brix) was determined as 72.65 ± 0.64/100 g (RA-500 Model Kyoto Electronics Manufacturing Co. Ltd., Kyoto, Japan).

Samples were stored at −20 °C until further analysis.

### 2.4. Pesticide Residue Analysis

Acetonitrile, glacial acetic acid, methanol, formic acid (with a quality of sufficient purity that is free of interfering compounds in LC/MS/MS) were obtained from Merck (Germany). Neat reference standards with purity >99% were obtained from Dr. Ehrenstorfer (Germany). Deionized pure water was used (Milli Q purification system, Merck, Germany) for the analysis. The standard solutions were prepared with 1% acetic acid acetonitrile and stored at −18 °C. Regarding chopping of the laboratory samples, each unit of the laboratory samples was divided into four, and two cross sections were homogenized until a final particle size of 2–3 mm was obtained (RechtGM 200, Haan, Germany). On the other hand, the laboratory sample coded as HJ was entirely homogenized, and the laboratory samples FJ and GP were not subjected to homogenization. Two analytical portions from each analytical sample were taken and stored for one month at −20 °C in capped polypropylene sample vials until further analysis was performed.

The QuEChERS (fast, easy, cheap, effective, robust, and safe) extraction method was applied [20]. The details of the method, including LC-MS/MS identification and equipment parameters, were provided by Acoglu and Yolci Omeroglu [2]. 

All household processes were repeated three times, and pesticide residue analyses including LC-MS/MS injections were performed in duplicates for each analytical sample. Results were presented as mean ± standard error.

### 2.5. Processing Factor

The processing factor (*P*_F_) can be calculated by Equation (1) [5,15]. A factor less than 1 or higher than 1 is an indication of a decrease or concentration, respectively.

(1) $$P_{F}=\frac{B\;}{RAC}$$

where *B* refers to the residue level in the processed orange samples (W, P, PU, FJ, HJ, and GP). When the amount of pesticide residue in the processed product (*B*) was lower than the limit of quantification of the method (LOQ), the processing factor was determined by replacing *B* with the LOQ level in the Equation (1). Consequently, the processing factor was expressed with an asterisk “<” [5,7,21].

The effect of processing on pesticide residue concentration was evaluated by the calculation of the reduction ratio or concentration ratio (*C*_R_) as given in Equation (2) [22];

(2) $$C_{R}\;\left(\%\right)=\frac{RAC-B}{RAC}\times 100$$

## 3. Results and Discussion

### 3.1. Reliability of the Analytical Method

The method used in the scope of the study was validated and approved by the Association of Official Agricultural Chemists (AOAC) as a standardized method [20]. Therefore, the method was verified at our laboratory conditions with a minimum requirement to obtain the provision of objective evidence that a given item fulfils specified requirements [23].

The standard analysis method applied in the scope of the study for the pesticide residue analyses was verified at 10 µg/kg to 60 µg/kg levels for high acid and water-containing foods and foods with high sugar and low water activity in accordance with the European SANTE/11312/2021 Guidance Document [24]. The following parameters were evaluated in the scope of the verification study: linearity, limit of quantification (LOQ), trueness (in terms of recovery), precision (repeatability and interim precision-intra-laboratory reproducibility), and measurement uncertainty.

The quantification and linearity of the method were evaluated during method verification study using matrix-matched calibration curves. Non-treated control orange samples were extracted as blank samples according to the extraction method described previously and the extracts were fortified with multi-standard working solutions at seven concentrations in a range between 2 µg/L and 80 µg/L. Moreover, during the analysis of the processed samples in the scope of the study (Table 1), multi-level calibration curves were constructed to cover the response of the pesticide residue in the sample at concentrations ranging between 10 µg/kg and 4000 µg/kg. The concentrations of pesticide active substances in samples were calculated in µg/kg using the weighted linear calibration curve functions with regression coefficients (R^2^) > 0.9999.

**Figure 2:**
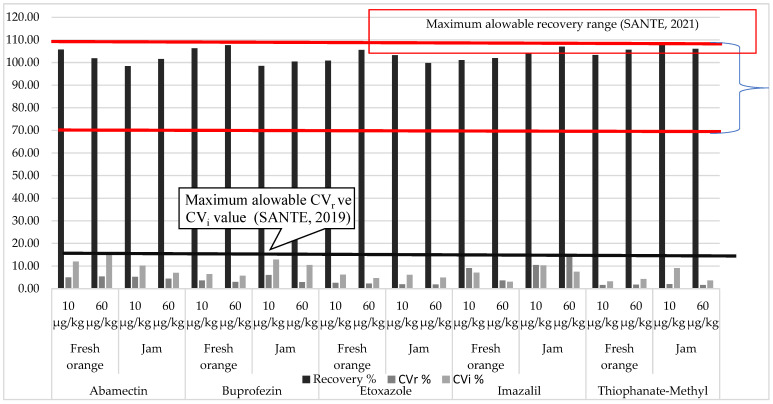
Method verification results (SANTE 2021).

As a result, mean recovery (%), repeatability (% CV_r_), and intra-laboratory reproducibility (% CV_i_) of the method ranged between 98.54–107.60%, 0.82–14.06%, and 1.56–16.23%, respectively (Figure 2). In addition, a blank sample was spiked with pesticides at the level of 10 µg/kg for each batch of analysis (*n* = 9), and the average recovery ratio was obtained in the range of 91% to 108%. LOQ was 10 µg/kg. The method performance parameters were compatible with the criteria set in the SANTE guideline [24].

Uncertainty is defined as a non-negative parameter characterizing the dispersion of the quantity values being attributed to a measurement based on the information used. There are two basic approaches to estimate measurement uncertainty, namely the bottom-up and top-down approaches. The top-down approach can be based on the method validation/verification and inter-quality-control laboratory dataset obtained within a laboratory. Combined measurement uncertainty arising from laboratory operations (*u*_Laboratory_) can be estimated from the square root of the quadratic sum of the random component (*u*_Rw_) and the systematic component (*u*_Bias_):

(3) $$u_{\mathrm{Laboratory}}\;=\sqrt{u_{\mathrm{Rw}}{}^{2}+u_{\mathrm{Bias}}{}^{2}}$$

**Figure 3:**
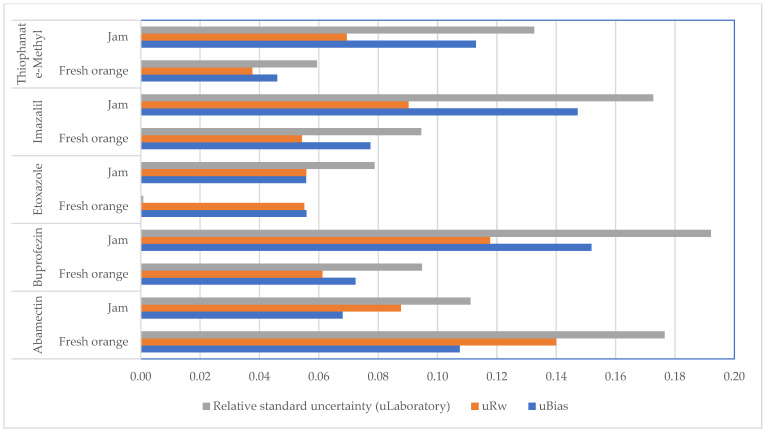
Uncertainty components (SANTE 2021).

*u*_Rw_ is the coefficient of variation of within-laboratory reproducibility which reflects all variation under routine conditions. *u*_Bias_ is the root mean square of the individual bias values and obtained from recovery studies [25,26]. As shown in Figure 3, the relative standard combined uncertainty arises from laboratory operations ranging between 0.1% and 19.21%, which corresponds to 0.2–38.42% of the expanded combined uncertainty at a 95% confidence level (k = 2). Those values comply with the maximum default relative expanded measurement uncertainty set as 50% in the European SANTE/11312/2021 Guidance Document [24].

### 3.2. Effect of Household Food Processing on Residue Content of Orange

The concentration of the residue in RAC should be higher than the limit of quantification (LOQ) of the analytical methods [15]. After the dipping of the orange samples into the commercial formulations in laboratory conditions, it was observed that the amount of pesticide residue in the treated orange samples (TC) ranged from 0.030 mg/kg to 3.753 mg/kg (Table 1), therefore the criteria set by OECD guideline was met in the scope of the study. Processing factors were calculated with Equation (1) and shown in Table 1.

#### 3.2.1. Washing (W)

Washing is the pretreatment in any type of food processing. It was observed that the washing process under tap water reduced the initial concentration of the residues in oranges by 26–84%, and processing factors ranged between 0.173 and 0.776. Consistently with our findings, other studies reported in the literature prevailed that pesticide residue decreased because of the washing of fruits and vegetables [2,27,28].

Li et al. [3] reported that washing of oranges with tap water reduced the residues of carbendazim, abamectin, imidacloprid, prochloraz, and cypermethrin by 43.6–85.4%. Another other study reported by Kwon et al. [29] revealed that the residues of chlorothalonil, oxadixyl, and thiophanate-methyl in tomatoes decreased by 92%, 52%, and 84%, respectively, after washing for 10 s. Acoglu and Yolci Omeroglu [2] investigated the effect of different washing agents on the fate of pesticides in orange. After washing oranges by dipping them into cold water (<10 °C) for 20 min, pesticide residues of abamectin, buprofezin, etoxazole, imazalil, and thiophanate-methyl decreased by 3–68%. In the current study, the additional hand rubbing operations eased the removal of pesticides during the washing of oranges.

After the washing step, the highest reduction (84%) was obtained for thiophanate-methyl, which has the lowest octanol/water partition coefficient (LogP) value among the other pesticides (Figure 1). The lowest reduction ratios (31–38%), achieved for abamectin, buprofezin, and etoxazole, can be attributed to their lower water solubility and higher LogP values compared to others. The octanol/water partition coefficient (LogP) of pesticides is the ratio of the solubility of a compound in octanol (a nonpolar solvent) to its water (a polar solvent) solubility [2]. Even though imazalil has a high water solubility (180 g/mL) 26% of the initial concentration was reduced after washing with tap water by hand. Romeh et al. [30] stated that the nature of the harvested crops was effective on the pesticide residue content of the final product. They demonstrated that pesticides can dry on the surface of the fruit and can be absorbed by the outer waxy structure. In this context, although imazalil has a systemic action and should diffuse through the inner part of the fruit, it deposited on the outer oily layer of the peel due to its high octanol–water coefficient as 3.82. In the same context, most of the studies demonstrated that pesticides with low LogP values can be easily washed away from the crop surface compared to the pesticides with high LogP values [2,11]. Polat and Tiryaki [28] observed that the reduction effects of different washing processes for capia pepper was related to the physicochemical properties of the pesticide (including mainly water solubility and an octanol–water coefficient). Similarly, Vass et al. [31] observed that 2% of the imazalil residue in lemon was reduced by washing with cold water.

#### 3.2.2. Peeling: Separation of Peel and Pulp

Peeling is the first step during the processing of many fruits and vegetables. It is an effective method to cut out the outer layers or skins of fruits and some vegetables and to reduce pesticide residues. It is also reported that chemical, mechanical, steam, or freezing peeling processes can provide a significant removal of residues depending on the chemical nature of the pesticides and environmental conditions [9,32]. 

In the scope of this study, it was concluded that the average initial concentration of the residues increased by 83% to 270% for obtaining the peel (P). As a result of the separation of the orange peels from the fruit pulp (PU), abamectin and etoxazole residues were not detected in the pulp, while a decrease was observed in the concentration of buprofezin, imazalil, and thiophanate-methyl by 57%, 73%, and 86%, respectively. In the same manner, processing factors for peel and pulp ranged between 1.40 and 4.83 and <0.120 and 0.423, respectively. In line with our observations, Yolci Omeroglu et al. [33] reported that the residue concentration of benomyl, which is a systemic fungicide, decreased by 41% to 83% by peeling. Li et al. [3] concluded that immidacloprid, carbendazim, abamectin, and cypermethrin residues were mostly distributed on peel of the orange. They reported that 7.5 to 17.9% of the initial residues diffused through the pulp of the fruit. Kwon et al. [29] observed that peeling process decreased the chlorothalonil, oxadixyl, and thiophanate-methyl concentration in tomato by 96%, 60%, and 93%, respectively.

After the peeling process, the pesticide residues remained on the peel layer of the fruit and mass transfer of the residues from peel to the pulp occurred at a lower diffusivity rate. This can be attributed to the physicochemical properties of the pesticides investigated in the scope of the study. Since thiophanate-methyl, etoxazole, and abamectin are contact and semi-systemic pesticides, they retained on the peel and did not diffuse through the pulp section of the fruit. In the same context, as a systemic pesticide imazalil was expected to diffuse completely through the pulp of the fruits, but its diffusion to the pulp section occurred on a limited scale. This can be attributed to its high octanol-water coefficient. Pesticides with high octanol–water coefficients (logP) (Figure 1) can be easily absorbed by the wax on the orange peel and cannot be removed from the peel easily [28,32]. Similarly, studies reported in the literature emphasize that the peeling process significantly decreased pesticide residues in fruits by peeling, and the reduction ratio was mainly based on the physicochemical properties of the active ingredients [27,32,34,35].

#### 3.2.3. Processing in to Fruit Juice (FJ)

The transition of pesticide residues from fruit to fruit juice depends on the distribution of the residue between peel and pulp in addition to their physicochemical properties. Residues are also reduced by the steps in the fruit juice production process, including clarification steps such as centrifugation or filtration [8,31].

As shown at Table 1, while abamectin and etoxazole residues were not detected in fruit juice, buprofezin, imazalil, and thiophanate-methyl residues decreased by 93%, 79%, and 63% respectively. Processing factor ranged between <0.120 and 0.363. Similarly, Li et al. [3] found that imidacloprid, abamectin, cypermethrin, and prochloraz residues decreased by 46.5%, 46.0%, 94.7%, and 81.0%, respectively, by obtaining orange juice from fresh oranges.

During the processing of oranges into fruit juice it was observed that highest reduction ratio was obtained for the pesticides with low water solubility (abamectin and etoxazole), while most of the residue concentration of the pesticides with higher solubility (thiophanate-methyl and imazalil) transferred into the juice. These findings can be attributed to the distribution of the pesticide residues between fruit peel/pulp and juice, depending on their water solubility [36].

#### 3.2.4. Frozen Storage of Grated Peels (GP)

Freezing is a commonly used food preservation method, as it slows down the chemical reactions that affect food quality, and retain flavor, texture, and nutritional quality of the final products better than other methods [31]. Orange peels can be stored as frozen forms to be evaluated as a by-product, to prolong its shelf life without any deterioration in its physicochemical structure, and can be used as an ingredient in pastry products.

In the scope of the study, pesticide residues in GP were significantly higher (*p* < 0.05) than the residue levels of the control orange samples (TC) (Table 1). Comparing the residue level of the frozen grated peels with the fresh peels (P), it was observed that there was a significant difference (*p* < 0.05), except for in abamectin. It can be related to the difference between the structure of the GP and P samples. GP was obtained by grating the top layer of the peel which did not contain any trace of the mesocarb section. As a result of a three-month storage periods, it was observed that pesticide residues in GP decreased significantly (*p* < 0.05). Therefore, it was concluded that the storage period affected the residue concentrations. Processing factors changed between 1.91 and 6.07, 1.780 and 5.33, 1.11 and 4.80, after the first, second, and third month of the frozen storage periods, respectively. Similarly, Ögüt et al. [37] reported that diazinon, parathion-methyl, captan, methidathion, cypermethrin, and deltamethrin residues in frozen cherries decreased during storage. The other studies reported in the literature supported these findings [37,38]

#### 3.2.5. Homemade Jam Processing (HJ)

Jams and marmalades are products in which fruits are made to be durable by high-heat treatment with the addition of sugar. As shown in Table 1, abamectin, etoxazole, and thiophanate-methyl residues were not detected in orange jam, while buprofezin and imazalil decreased significantly by 90% and 95% considering their initial levels. Consequently, processing factors were obtained (between 0.05 and <0.330). Within the scope of the study, in order to process orange jam, boiling was carried out as a pre-treatment step. During the boiling step, boiling water was replaced with fresh water three times. At the last step, cooking was carried out by adding fresh water and sugar to the fruits. Therefore, the reduction of pesticide residue levels in the jam could be attributed to the removal of water-soluble pesticides with boiling water [8,32,39,40], evaporation, decomposition, heat degradation, time, and the temperature of the heat treatment process applied for cooking [41,42]. Hendawi et al. [43] reported that imidacloprid residue decreased by about 15% with the processing of strawberries, in which the boiling of the fruit and removal of boiling water was not applied, in contrast to our study. Lozowicka et al. [42] revealed that thiophanate-methyl residue decreased by a ratio of 82% and difenoconazole decreased by 29% during the production of black currant jam. Therefore, it can be stated that there may be a difference in the reduction rate according to the jam cooking technique and the physicochemical structure of the pesticide residue in the product.

## 4. Conclusions

The fate of pesticides during various household and industrial processes depends on the type of processing methods, the physicochemical properties of the pesticides, and the nature of the product. Pesticides with high octanol–water coefficients were absorbed by the wax of the orange peel, therefore they remained on the peel and could not easily be removed by the washing step. Moreover, pesticides with lower water solubility did not diffuse easily through the fruit juices from the pulp section of the fruit. The processing factor was greater than 1 for the separation of the orange peel and less than 1 for the washing step and jam and fruit juice production. The compliance of residues in processed commodities with MRLs set for RAC should be assessed by taking into account the processing factor values. Furthermore, processing factors are essential to estimate residues in processed commodities for conducting dietary risk assessment. Therefore, it is essential to reveal the processing factors with scientific studies in order to make legal arrangements. It should be indicated that the dipping of the crops into commercial formulations of the pesticides under laboratory conditions does not reflect real processing effects. Therefore, to obtain more accurate processing factors, in future studies field treatment of the crops should be applied. In field-treated crops, pesticides may penetrate into different sections of the plants based on the pre-harvest interval. Absorption and translocation of the pesticide through the crops may affect the fate of the pesticide throughout the processing. Consequently, this study can be taken as a case study and more extensive studies should be carried out for different type of foods and different type of processing methods with field-treated crops.

## Figures and Tables

**Table 1:** Effect of household processing of orange on the concentration of pesticide residue and processing factors (*n* = 3).

	Abamectin		Buprofezin		Ethoxazole		Imazalil		Thiophanate-Methyl	
Process	C (mg/kg)	*P*_F_	C (mg/kg)	*P*_F_	C (mg/kg)	*P*_F_	C (mg/kg)	*P*_F_	C (mg/kg)	*P*_F_
Raw agricultural commodity (RAC)	0.030 ± 0.004	-	0.189 ± 0.047	-	0.082 ± 0.025		2.113 ± 0.475		0.110 ± 0.030	
Washing	0.020 ± 0.003	0.663 ± 0.115	0.136 ± 0.023	0.720 ± 0.121	0.050 ± 0.001	0.616 ± 0.011	1.643 ± 0.271	0.776 ± 0.127	0.019 ± 0.003	0.173 ± 0.028
Peeling: Separation of peel	0.080 ± 0.012	2.68 ± 0.42	0.650 ± 0.091	3.43 ± 0.48	0.396 ± 0.025	4.83 ± 0.307	2.963 ± 0.366	1.40 ± 0.176	0.404 ± 0.041	3.67 ± 0.37
Peeling: Separation of pulp	<LOQ	<0.330	0.080 ± 0.014	0.423 ± 0.076	<LOQ ^1^	<0.120	0.453 ± 0.011	0.216 ± 0.005	0.015 ± 0.005	0.146 ± 0.005
Fruit juice	<LOQ	<0.330	0.037 ± 0.001	0.200 ± 0.001	<LOQ ^1^	<0.120	0.580 ± 0.001	0.270 ± 0.001	0.039 ± 0.316	0.363 ± 0.063
Homemade jam	<LOQ	<0.330	0.019 ± 0.005	0.103 ± 0.028	<LOQ ^1^	<0.120	0.105 ± 0.008	0.050 ± 0.001	<LOQ ^1^	<0.090
Frozen storage of grated peels (1st month)	0.090 ± 0.017	2.83 ± 0.58	0.840 ± 0.036	4.60 ± 1.04	0.466 ± 0.030	6.07 ± 1.92	3.866 ± 0.075	1.90 ± 0.487	0.520 ± 0.051	4.88 ± 0.29
Frozen storage of grated peels (2nd month)	0.069 ± 0.011	2.32 ± 0.39	0.686 ± 0.025	3.62 ± 0.13	0.436 ± 0.005	5.33 ± 0.08	3.753 ± 0.254	1.78 ± 0.121	0.486 ± 0.127	3.53 ± 0.77
Frozen storage of grated peels (3rd month)	0.032 ± 0.011	1.11 ± 0.43	0.533 ± 0.020	2.81 ± 0.11	0.393 ± 0.152	4.80 ± 0.19	2.846 ± 0.06	1.35 ± 0.02	0.390 ± 0.085	3.26 ± 1.73

^1^ LOQ: limit of determination, 0.01 mg/kg, residue amount (B) is taken as 0.010 mg/kg when calculating the processing factor for these processes (Scholtz vd., 2017).

## Data Availability

Data is contained within the article.
